# PROF BARRIE JONES

**Published:** 2009-12

**Authors:** Gordon Johnson

Dear Editor,

It was with great sadness that we learnt of the death of **Prof Barrie Jones** on 19 August 2009 in Tauranga, New Zealand, aged 88.

He was admired worldwide for his work in developing the concept of prevention of blindness and for establishing the International Centre for Eye Health (publishers of the Community Eye Health Journal) in 1981. He was in fact responsible for encouraging the launch of the first issues of this journal. However, your readers may not be aware of his major contributions, earlier in his career as first clinical professor of ophthalmology in the University of London, to the science and management of corneal and external eye disease.

Barrie first addressed the range of virus infections of the eye seen in London, writing about adenovirus infection and corneal involvement by the vaccinia virus. He conducted much laboratory research on herpes simplex infection, and randomised trials of interferon, idoxuridine, trifluorothymidine, adenine arabinoside, and ultimately acyclovir. A rational approach was developed to the management of different stages of herpetic keratitis, including mechanical debridement, when to use corticosteroids, and the role of corneal grafting.^1^

**Figure:**
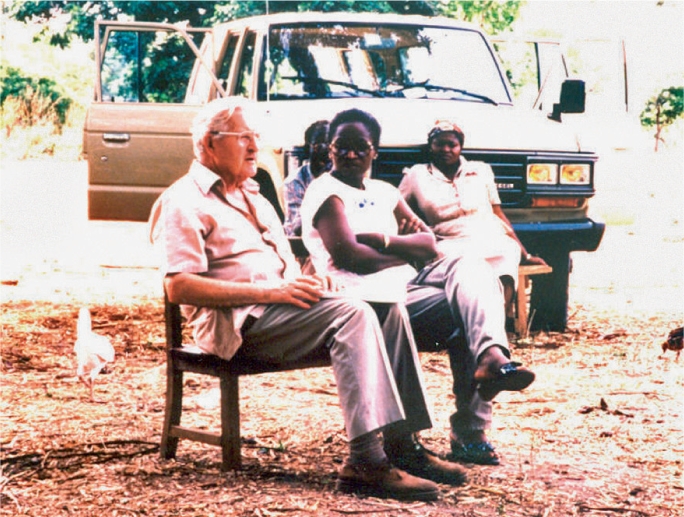
Barrie Jones, after his retirement, discussing the progress of the clinical trial of ivermectin with Prof Adenike Abiose. NIGERIA

Although a much less common cause of keratitis than in tropical countries, fungi nonetheless caused serious corneal infections in London. Barrie and his colleagues cultivated and tested the sensitivity of every fungus isolate to many different potential new drugs. He emphasized that the variations in sensitivity within each species were so great that it was necessary to base rational therapy on the results of sensitivity testing of each patient's own fungus.^2^

Barrie Jones was widely known as an authority on many aspects of trachoma. He developed methods of isolation, culture, and laboratory diagnosis. In extensive field studies in Tunisia, Iraq, and Iran he elucidated the seasonal dynamics of transmission of blinding hyperendemic trachoma, recognising the importance of multicyclic re-infection and the role of flies in transmission in these countries. He carried out trials of early potential vaccines and of chemotherapy.^3^

He investigated and wrote about an astonishing range of other corneal conditions, including infections by amoebae, vernal and other allergic types of keratoconjuntivitis, Thygeson's superficial punctate keratitis, pemphigoid, and dry eye syndromes. In the operating theatre, he pioneered new surgery of the lacrimal canaliculi and duct and improved the techniques, postoperative management, and outcomes of corneal grafting.

When, around 1980, he turned the full energy of his thinking to the questions of blindness prevention, it was informed by this rich background of laboratory and clinical experience.
